# A typical multisite invasive infection caused by hvKP: A case report and literature review

**DOI:** 10.1097/MD.0000000000032592

**Published:** 2022-12-30

**Authors:** Jia Liu, Mingying Dai, Qiang Sun, Wei Fang

**Affiliations:** a College of Medicine, Qingdao University, Qingdao, China; b Department of Critical Care Medicine, The Affiliated Hospital of Qingdao University, Qingdao, China; c Department of Critical Care Medicine, Shandong First Medical University Affiliated Province Hospital, Jinan, China.

**Keywords:** diabetes, hypervirulent *K. pneumoniae* (hvKP), liver abscess

## Abstract

Hypervirulent K. pneumoniae (hvKP) is a new variant of K. pneumoniae that can caused metastatic spreading by blood, including Splenic abscess, endogenous endophthalmitis, purulent meningitis. This report described a case of A typical multisite invasive infection caused by hvKP and carry out a historical review of the literature.

**Patient concerns:**

A 55-year-old man was referred to our hospital due to liver abscess. *Diabetes* was found during this hospitalization. Because of glycemic was uncontrolled, splenic abscess, endogenous endophthalmitis and purulent meningitis occurred during subsequent treatment.

**Diagnoses:**

We made s diagnosis of liver abscess and *invasive K. pneumoniae* liver abscess syndrome through generation sequencing and imaging features.

**Interventions and outcomes:**

The patient recovered and was subsequently discharged after mechanical ventilation, continuous renal replacement therapy, *laparoscopic exploration* and various antimicrobials.

**Lessons:**

HvKP are very aggressive and can disseminate to multiple sites, especially in patient who have *diabetes, which is a treatment challenge for* clinician.

## 1. Introduction

*Klebsiella pneumonia* is a frequent opportunistic pathogen which widely spread in our respiratory tract, urinary tract, gastrointestinal tract, etc. Current evidence shows the existence of 2 major types of hypervirulent *K. pneumoniae* (hvKP): classic *K. pneumoniae* and hvKP. hvKP was a new variant of *K. pneumoniae*. Different from classic *K. pneumoniae*, hvKP exhibit severe invasiveness that can cause metastatic spreading by blood, including splenic abscess, endogenous endophthalmitis, and purulent meningitis. Here we report a typical multisite invasive infection caused by hvKP to provide new ideas for clinical treatment of this infection.

## 2. Case report

**Figure 1.**
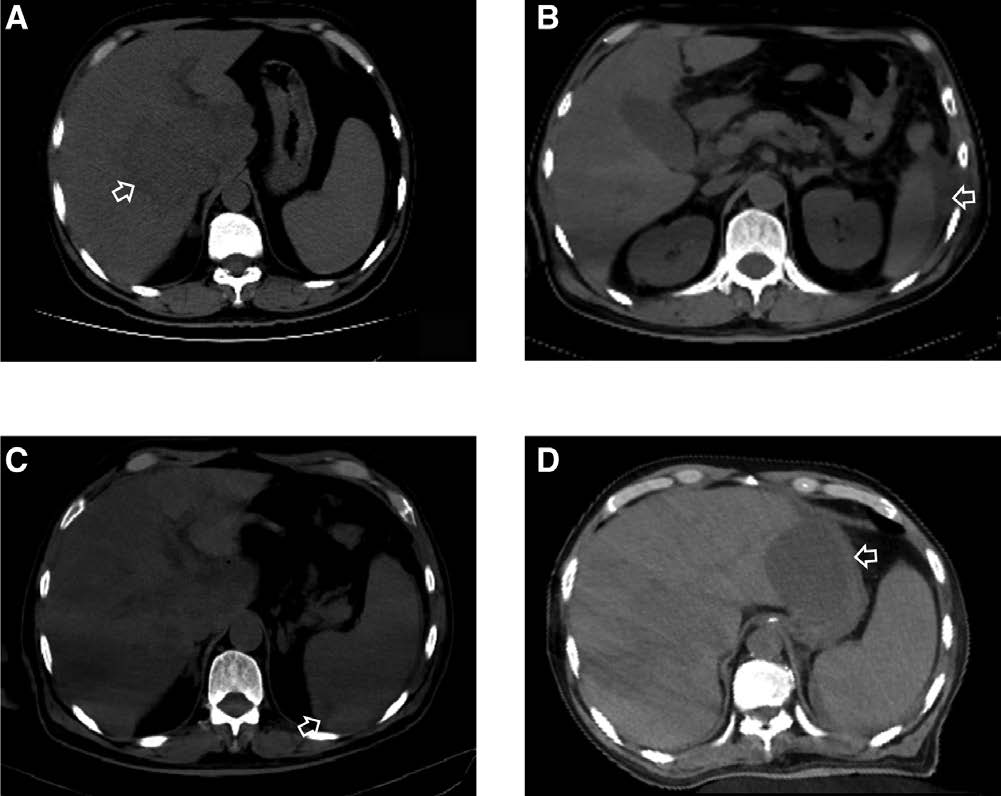
(A) Chest CT showed liver abscess before admission. (B and C) After 4 days in ICU, perisplenic and splenic abscess occurred. (D) After 8 days in ICU, abdomen CT showed cystic low-density shadow around the gastric wall, considering abscess liquefaction. CT = computed tomography, ICU = intensive care unit.

**Figure 2.**
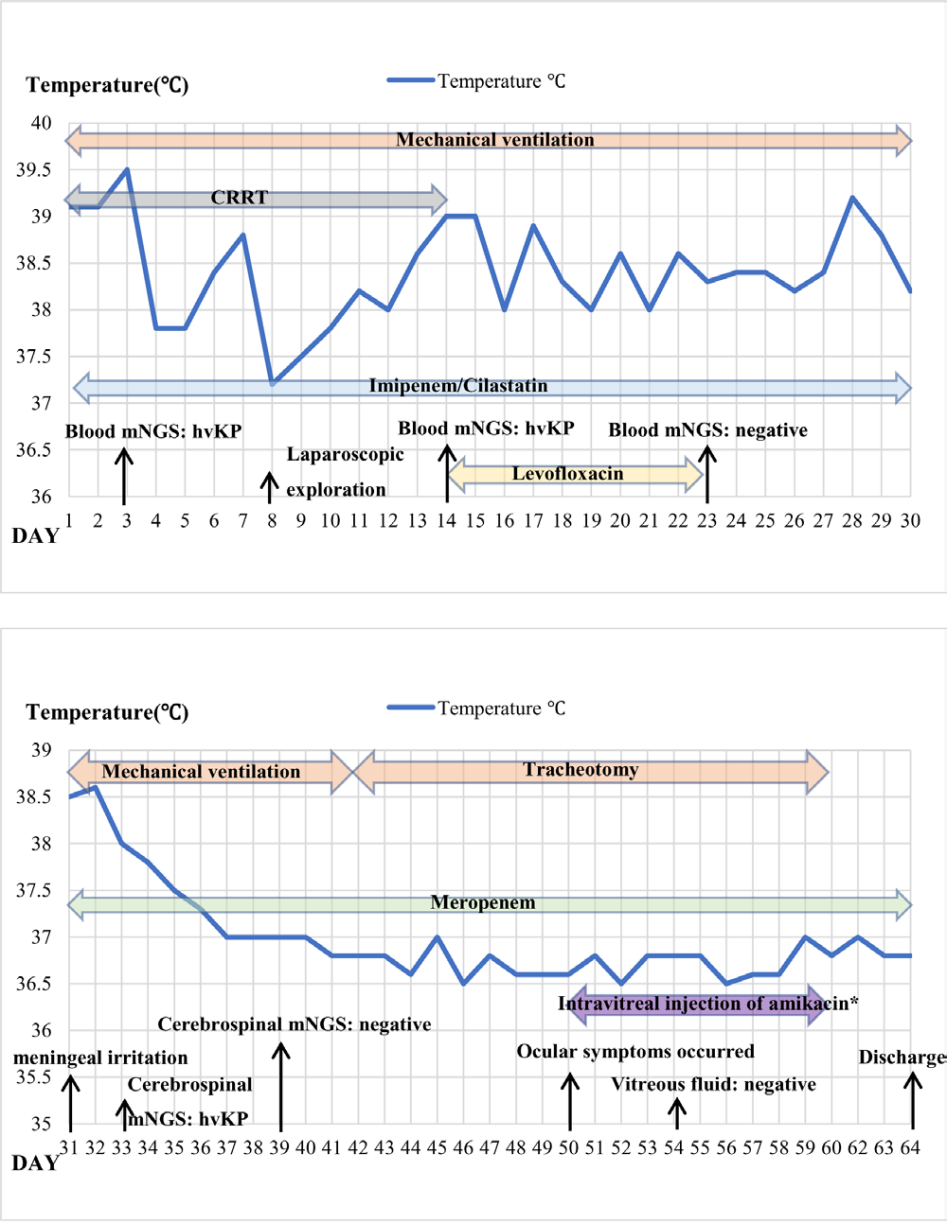
A brief summary of the patient’s medical history in ICU. She was treated for 64 days in total. Antibiotics doses: imipenem/cilastatin, 2 g every 6 hours, levofloxacin, 0.5 g every day, meropenem, 2 g every 8 hours.* Intravitreal injection of amikacin, qod, meanwhile, levofloxacin and tobramycin eye drop, q2h. ICU = intensive care unit, q2h = every 2 hours.

**Figure 3.**
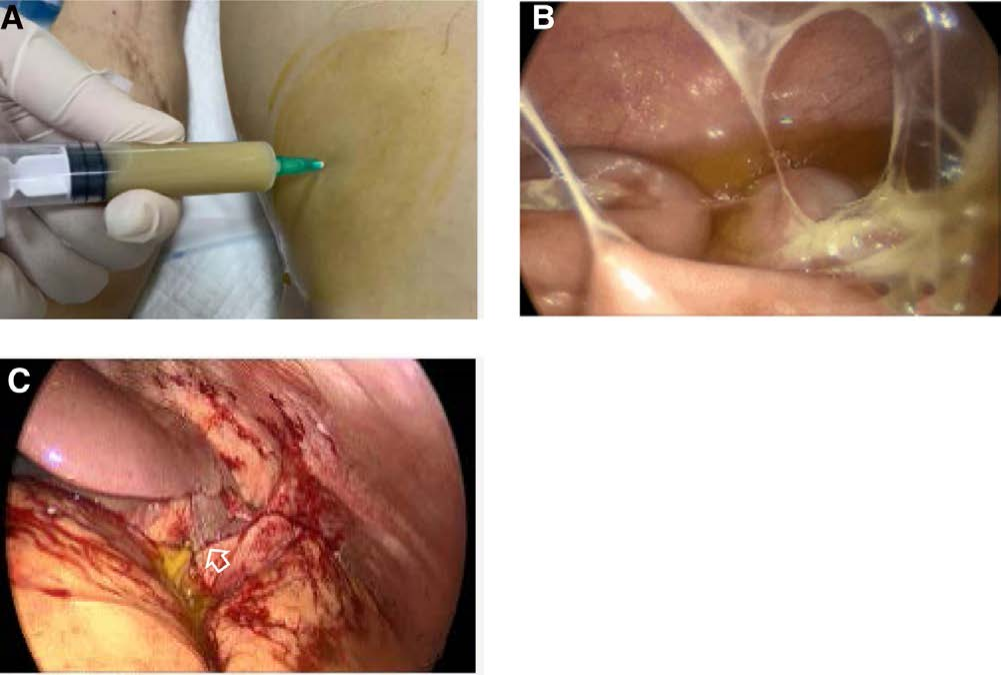
(A) A diagnostic abdominal puncture revealed yellow turbid pus. (B and C) Laparoscopic exploration showed purulent ascites in the abdominal cavity, adhesion of the omentum and part of the small intestine to the abdominal wall, and a 10-cm diameter cystic cavity in the visceral surface of the left external lobe of the liver with a large amount of yellow purulent fluid.

**Figure 4.**
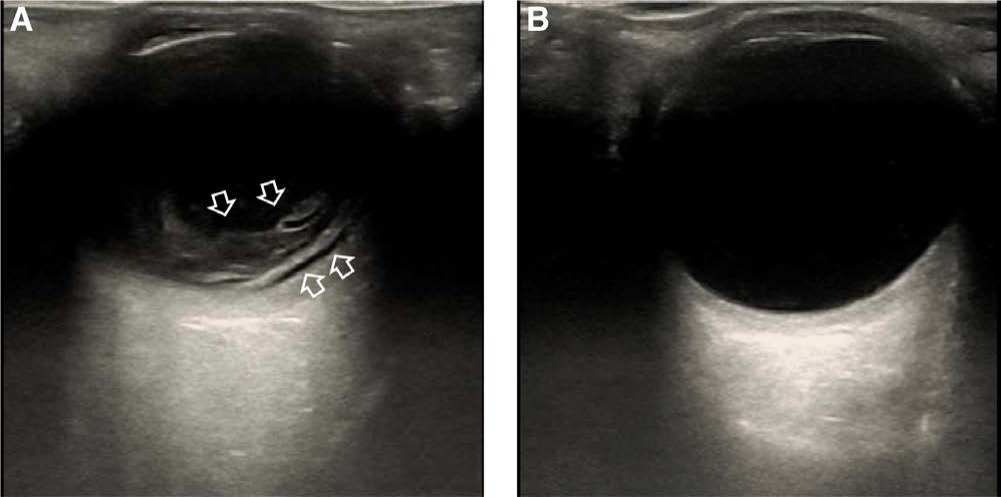
(A) Bedside ultrasonography showed numerous acoustic shadows in the vitreous cavity and retinal detachment of the right eye; (B) the left eye was normal.

A 55-year-old man was diagnosed with liver abscess (Fig. 1A) in Hepatopancreatobiliary on May 6, 2022. He didn’t know he had type 2 diabetes mellitus until this admission. After 3 days, he was transferred to the intensive care unit (ICU) due to septic shock. In ICU, he was treated with mechanical ventilation, continuous renal replacement therapy, surgery, and various antimicrobials (Fig. 2). At the time of ICU admission, the patient was unconscious and had a fever of 39.1°C. Laboratory tests showed the procalcitonin level of >100ng/L, blood sugar level of 28.1 mmol/L, and glycated hemoglobin level of 11.2%. Because of abdominal cavity effusion, we performed abdominal puncture and drainage (Fig. 3A). We took bacteriological evidence at the first time and used imipenem/cilastatin to anti-infective treatment. After few days, the blood and abdominal puncture fluid metagenomics next generation sequencing showed hvKP. Splenic abscess occurred by ICU day 3(Fig. 1B and C); we also did puncture and drainage. On day 8 in ICU, we performed laparoscopic exploration (Fig. 3B and C) because the abdominal contrast-enhanced computed tomography showed stomach walls with peripheral hypodense rim and takes on a cystic appearance (Fig. 1D). And 3 drains were indwelled in right inferior phrenic, pelvic, and abscess cavity. Everything is changing for the better. A month after the laparoscopic surgery, the patient presented with nuchal rigidity, and changed in mental status, cerebrospinal fluid culture also showed hvKP, the antibiotic was changed to Meropenem. After 50 days in ICU, we stumbled upon the fact that the patient’s right visual acuity had decreased light perception. However, no obvious abnormality was found in the appearances of the right eye. B-mode ultrasonography showed numerous acoustic shadows in the vitreous cavity and retinal detachment (Fig. 4) that confirmed the diagnosis of endogenous endophthalmitis. With the help of ophthalmologists, amikacin was used to treat endophthalmitis by intravitreal injection. Meanwhile, the treatment also consisted of the local application of levofloxacin and tobramycin. Of note, the result of vitreous fluid was negative. Following 13 days of therapy, vitreous opacities were alleviated. Given the patient’s body temperature was normal, blood cultures and cerebrospinal fluid culture became negative, physical examination revealed no signs of meningeal irritation. The patient was allowed to discharge and was followed up in the ophthalmology clinic.

## 3. Discussion

**Figure 5.**
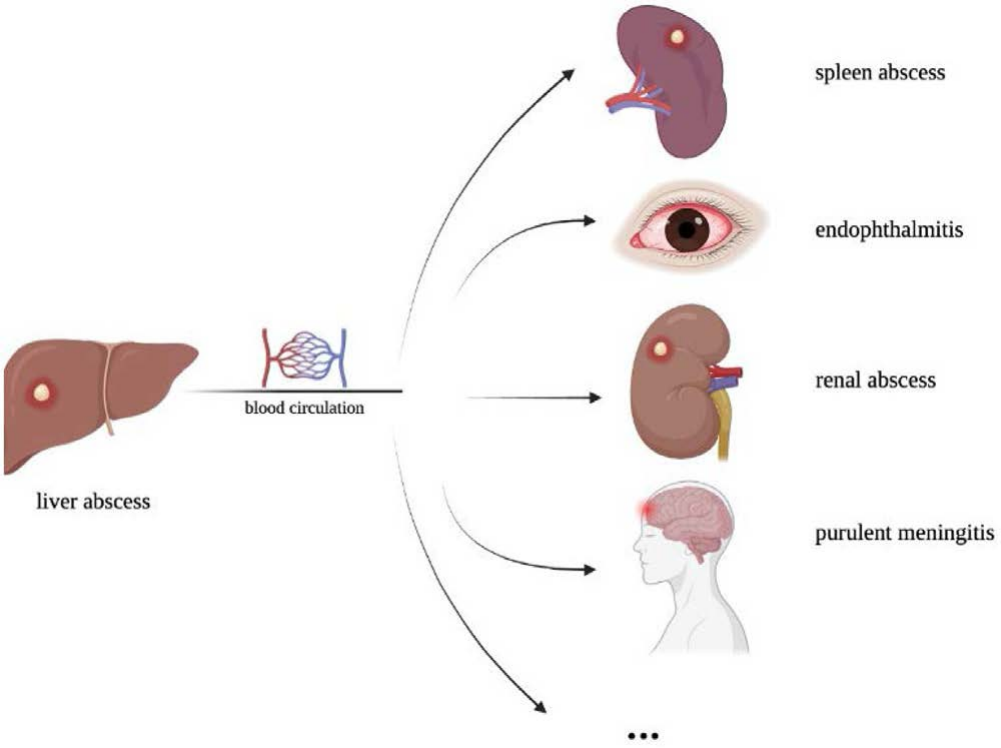
hvKP exhibit severe invasiveness that caused metastatic spreading by blood, including splenic abscess, endophthalmitis, renal abscess, endogenous endophthalmitis, called IKLAS. hvKP = hypervirulent *Klebsiella pneumonia*, IKLAS = invasive *K. pneumoniae* liver abscess syndrome.

*K. pneumonia* is one of the most isolated gram-negative bacteria. HvKP, a new type of *K. pneumoniae*, was first initiated in 1986 in Taiwan, China.^[1]^ But the definition of hvKP is inconclusive. With the developments of molecular biology technology, the specific genes which are located on the virulence plasmid (e.g.,: peg-344, iutA, iuc, and rmpA) are considered the best marker of hvKP in laboratory.^[2]^ Nowadays, hvKp infections demonstrate an increasing trend. We think hvKP have 2 particularly typical characteristics via review of the literature, firstly, hvKP infections are more common in immunocompetent Asian male.^[3]^ Evidence showed that Caucasians who encounter these populations or travel to Asia have a significantly higher risk of contracting hvKP. Some scholars speculated that this phenomenon might contribute to geographical region and race.^[4]^ In addition, hvKP have a strong ability of invasion that cause infections at multiple sites by blood called invasive *K. pneumoniae* liver abscess syndrome (IKLAS) (Fig. 5).^[5]^ Some studies have shown that the occurrence of IKLAS increased with poor glycaemic control. This patient fits the above characteristics and is a very typical case of IKLAS caused by hvKP infection.

The incidence of endophthalmitis in patients with hvKP liver abscess ranges from 3.4 to 12.6% and has been on the rise in recent years.^[6]^ This infection has insidious onset and rapid development, often leading to irreversible visual impairment. Previous diabetes is one of the important causes of endophthalmitis in patients with hvKP liver abscess. Compared with normal people, the risk of endophthalmitis in such patients is 3.6 to 11 times higher. The level of blood glucose control is also related to the invasiveness of hvKP. Studies have found that *K. pneumoniae* liver abscess with hemoglobin A1C >9% has a significantly increased risk of invasive infection, which experts speculate may be related to the increased vascular permeability caused by diabetes.^[7]^ Intraocular fluid (aqueous humor, vitreous humor) culture is considered the gold standard in the diagnosis of endogenous endophthalmitis. In this case, the result of intraocular fluid culture was negative, we think that may be associated with systemic antibiotics appliance, the impact of sampling location must not be overlooked either. Combined with his medical history, it is not difficult to draw the diagnosis of endogenous endophthalmitis, so we immediately took intraocular drug injection; it is important to note that endophthalmitis occurred after the treatment of liver abscess improved, which was related to the patient’s previous blood glucose control. Therefore, for prediabetic individuals, we should dynamically monitor their visual level and regularly do ocular-ultrasound to achieve early identification and diagnosis.

Purulent meningitis, another serious disease caused by hvKP invasion, has a high fatality rate.^[8]^ This neurological sequela is irreversible and requires early identification. Particularly, patients who are in an unconscious state or used artificial airway need careful daily physical examination. Performing computed tomography, ultrasound and other imaging examination, if necessary. It is for this reason that the meningitis is found at the first time and avoid the occurrence of neurological sequelae.

In conclusion, hvKP infection have features of being highly invasive, widely spread and atypical, which requires aggressive systemic search abscesses. Strict control blood glucose is also important. Once the diagnosis is made, sensitive antibiotics should be applied in time. When IKLAS is found, the relevant departments should be consulted for combined treatment.

## Author contributions

JL drafted the manuscript and collected the clinical data. MD, QS and WF helped to draft the manuscript.

**Data curation:** Jia Liu.

**Formal analysis:** Mingying Dai.

**Methodology:** Jia Liu, Qiang Sun, Wei Fang.

**Writing – original draft:** Jia Liu.

**Writing – review & editing:** Wei Fang.
